# Bioassay-Guided Antidiabetic Study of *Phaleria macrocarpa* Fruit Extract

**DOI:** 10.3390/molecules17054986

**Published:** 2012-04-30

**Authors:** Rabyah B. Ali, Item J. Atangwho, Navneet Kaur, Omar Saad Abraika, Mariam Ahmad, Roziahanim Mahmud, Mohd Z. Asmawi

**Affiliations:** 1 School of Pharmaceutical Sciences, Universiti Sains Malaysia, Minden 11800, Penang, Malaysia; Email: rabyah2010@yahoo.com (R.B.A.); mariam@usm.my (M.A.); amzaini@usm.my (M.Z.A.); 2 Department of Biochemistry, College of Medical Sciences, University of Calabar, P.M.B. 1115, Calabar, Nigeria

**Keywords:** *Phaleria macrocarpa*, antidiabetic, plasma insulin, medicinal plant, mangiferin

## Abstract

An earlier anti-hyperglycemic study with serial crude extracts of *Phaleria macrocarpa* (PM) fruit indicated methanol extract (ME) as the most effective. In the present investigation, the methanol extract was further fractionated to obtain chloroform (CF), ethyl acetate (EAF), *n*-butanol (NBF) and aqueous (AF) fractions, which were tested for antidiabetic activity. The NBF reduced blood glucose (*p* < 0.05) 15 min after administration, in an intraperitoneal glucose tolerance test (IPGTT) similar to metformin. Moreover, it lowered blood glucose in diabetic rats by 66.67% (*p* < 0.05), similar to metformin (51.11%), glibenclamide (66.67%) and insulin (71.43%) after a 12-day treatment, hence considered to be the most active fraction. Further fractionation of NBF yielded sub-fractions I (SFI) and II (SFII), and only SFI lowered blood glucose (*p* < 0.05), in IPGTT similar to glibenclamide. The ME, NBF, and SFI correspondingly lowered plasma insulin (*p* < 0.05) and dose-dependently inhibited glucose transport across isolated rat jejunum implying an extra-pancreatic mechanism. Phytochemical screening showed the presence of flavonoids, terpenes and tannins, in ME, NBF and SFI, and LC-MS analyses revealed 9.52%, 33.30% and 22.50% mangiferin respectively. PM fruit possesses anti-hyperglycemic effect, exerted probably through extra-pancreatic action. Magniferin, contained therein may be responsible for this reported activity.

## 1. Introduction

Diabetes mellitus is a major public health problem already at epidemic proportions globally. The world prevalence of diabetes among adults (aged 20–79 years) was estimated as 6.4%, affecting 285 million adults, in 2010, and is expected to increase to 7.7%, and 439 million adults by 2030. Between 2010 and 2030, there is a projected 69% increase in numbers of adults with diabetes in developing countries and a 20% increase in developed countries [1]. This increased incidence has already been reported in Asia and Malaysia in particular. For instance, a ten year consecutive survey carried out by the National Health and Morbidity Surveys I, II and III in Malaysia, respectively, reported diabetes prevalence among adults of 6.3% in 1986, 8.3% in 1996 and 11.6% in 2006 [2]. 

This ugly trend has necessitated increased exploration and exploitation of alternative therapeutic and management measures, particularly traditional herbal remedies. Lead information from traditional medicine and also scientific research with medicinal plants have reported maximum therapeutic efficacy, yet with increased safety margins, since most of these plants have formed part of the human foods for generations [3]. More so, diabetes being a syndrome with multi-faceted etiologies requires a multi-modal therapeutic approach capable of addressing simultaneously several targets; a holistic approach scarcely available in conventional therapy. The conventional therapeutic approaches mainly involve drugs that enhance insulin secretion or signaling, as well as inhibitors of endogenous glucose production [4]. Herbal medicine is thought to provide comparative advantage by reason of the diverse secondary metabolites present. However, adequate research on these medicinal plants beyond screening for biological activity, should be conducted with the aim to systematically standardize and develop them into natural products or dosage forms which would effectively complement or supplement existing conventional measures [5,6] as well as follow quality assessment and evaluation guidelines [7]. 

In line with the foregoing objective, the present study, using an ethnomedical-based drug discovery program, evaluated the antidiabetic activity of fruits of *Phaleria macrocarpa* (PM) used in the traditional health system of the Indonesians and lower course of Malaysia, as an effective remedy and management for diabetes mellitus and other ailments such as liver diseases, vascular problems, cancer, high blood pressure, rheumatism and acne, *etc.* [8,9]. An earlier hypoglycemic and anti-hyperglycemic screening of polarity graded extracts (successive extraction using petroleum ether, methanol and water) revealed that of the three extracts, methanol extract exhibited the most potent glucose lowering activity and effect on plasma insulin (unpublished data). The current investigation in furtherance of that previous study evaluated a bioassay-activity guided antidiabetic study of the active methanol extract of PM fruit pericarp. 

## 2. Results and Discussion

### 2.1. Results

#### 2.1.1. Effect of Methanol Fractions of PM in Glucose Tolerance Test

**Figure 1:**
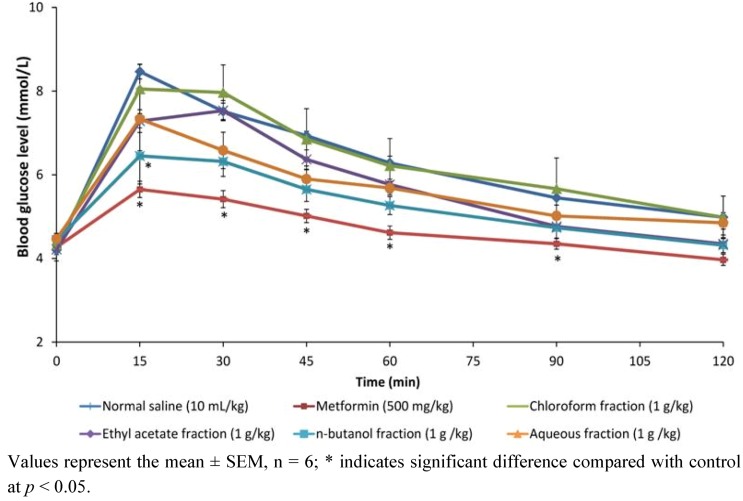
Effect of oral administration of chloroform, ethyl acetate, *n*-butanol and aqueous fractions of *P. macrocarpa* fruit (1 g/kg each) and metformin on blood glucose levels after an intraperitoneal glucose load (1 g/kg).

Figure 1 shows the effects of chloroform, ethyl acetate and aqueous fractions and metformin on blood glucose levels of non-diabetic rats following an oral glucose challenge. Of the four fractions-treated groups, rats treated with *n*-butanol fraction were the most responsive to the glucose challenge, as the glucose levels significantly decreased (*p* < 0.05) just 15 min after the glucose load similar to metformin the reference drug. Although not statistically significant, this decrease caused by the *n*-butanol fraction was also sustained in tandem with metformin until end of experiment. The effect of chloroform, ethyl acetate and aqueous fractions were not significant compared to both negative and positive controls.

#### 2.1.2. Effects of Methanol Fractions of PM on Blood Glucose of Diabetic Rats during and at the End of a 12-Day Treatment

**Figure 2:**
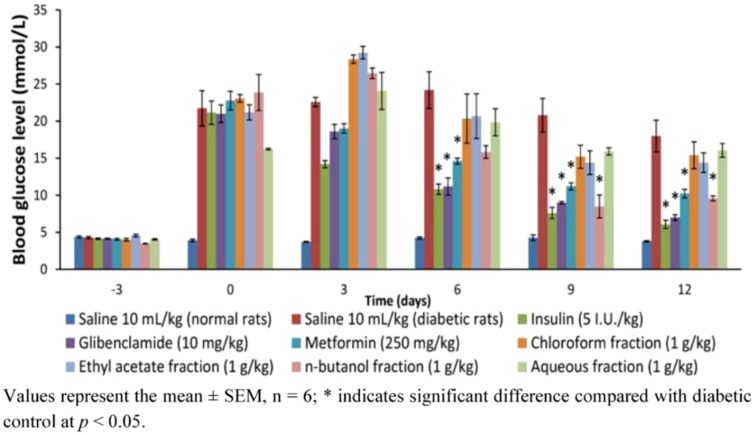
The effect of 12-day repeated administration of chloroform, ethyl acetate, *n*-butanol and aqueous fractions of PM fruits (1 g/kg) on fasting blood glucose level of Streptozotocin-induced diabetic rats measured on days 3, 6, 9 and 12.

The effect of 12-day repeated treatment of methanol extract fractions (chloroform, ethyl acetate and aqueous fractions) and reference drugs (metformin, glibenclamide and insulin) on fasting blood glucose of diabetic rats during and at end of study is shown on Figure 2. It is clear from the result that whereas the reference drugs significantly reduced blood glucose from day 6 and sustained it thereafter (*p* < 0.05), of the four fractions only the *n*-butanol fraction could decrease blood glucose from day 9 and sustain it to end of the experiment (*p* < 0.05). Compared with the concentration at outset of treatment, the *n*-butanol fraction was seen to decrease glucose after 12 days treatment by 66.67% (*p* < 0.05); to compare favorably with metformin (51.11%), glibenclamide (66.67%) and insulin (71.43%), and was hence considered to be the most active fraction. 

#### 2.1.3. Effects of *n*-Butanol Sub-Fractions I and II on Glucose Tolerance in Non Diabetic Rats

**Figure 3:**
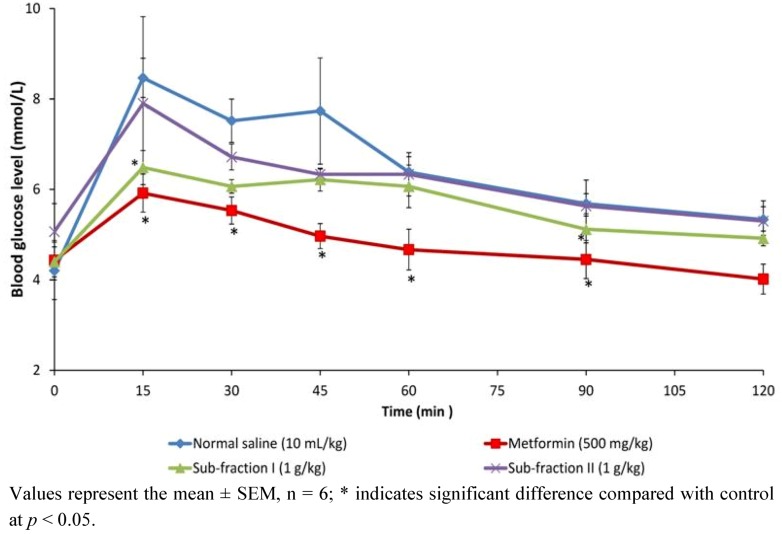
The effect of oral administration of sub-fractions I and II of *P. macrocarpa* fruit (1 g/kg) on blood glucose levels after an intra-peritoneal glucose load (I g/kg) in normal rats.

The effect of sub-fractions of *P. macrocarpa* on intrapritoneal glucose tolerance test in normal rats is shown in Figure 3. Glucose levels in the three treatment groups and control reached their respective peaks (Peak Blood Glucose, PBG) at 15 min after oral loading. Sub-fraction I only, caused significant reduction in PBG similar to metformin when compared to control (*p* < 0.05), and maintained it through the 45th and 90th min. Sub-fraction I therefore had a better response to glucose challenge in normal rats compared to sub-fraction II which failed to affect measured blood glucose significantly.

#### 2.1.4. Effect of Single Dose of Sub-Fractions I and II on Blood Glucose of Diabetic Rats

**Figure 4:**
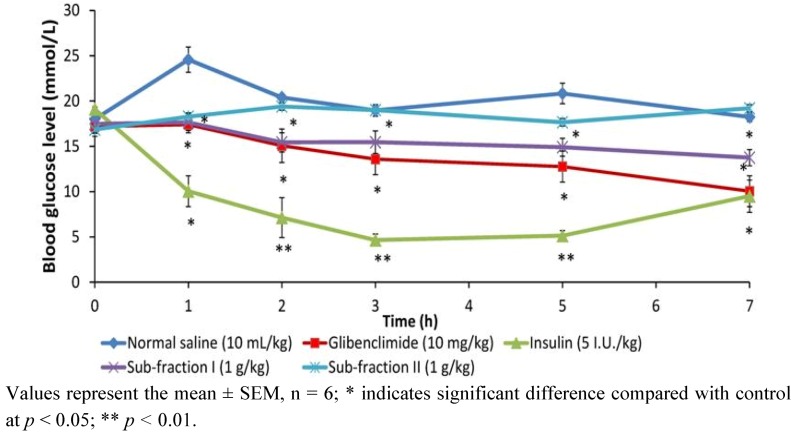
The effect of single dose (1 g/kg) administration of sub-fractions (I and II) of *P. macrocarpa* fruit on blood glucose levels of diabetic rats measured over 7-hour period.

To determine the time-dependent effect of the sub-fractions, a single dose (1 g/kg) was administered to diabetic rats and blood glucose intermittently monitored with a glucose meter for 7 h compared to insulin and glibenclamide (Figure 4). Compared to diabetic control, sub-fraction I was seen to lower blood glucose from the first one hour (*p* < 0.05), and this reduction was maintained throughout the seven-hour period of the experiment similar to glibenclamide. Administered insulin also lowered the blood glucose within this duration, but however to a higher extent than the sub-fraction I and glibenclamide between the first and fifth hour (*p* < 0.05). Similar to the result of glucose tolerance test, sub-fraction II did not cause significant effect on blood glucose.

#### 2.1.5. Effect of 12-Day Repeated Treatment with Active Extract, Fraction and Sub-Fraction on Measured Blood Glucose and Plasma Insulin Levels of Diabetic Rats

**Figure 5:**
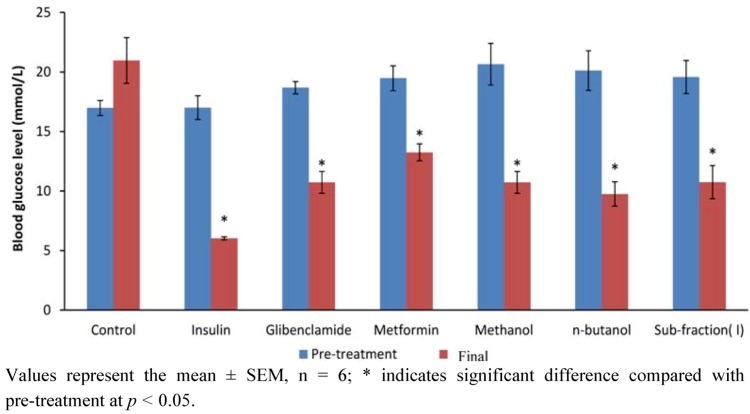
Fasting blood glucose concentrations of diabetic rats before and after treatment with methanol extract, *n*-butanol fraction and sub-fraction I and reference drugs (metformin, glibenclamide and insulin) for 12 days.

**Figure 6:**
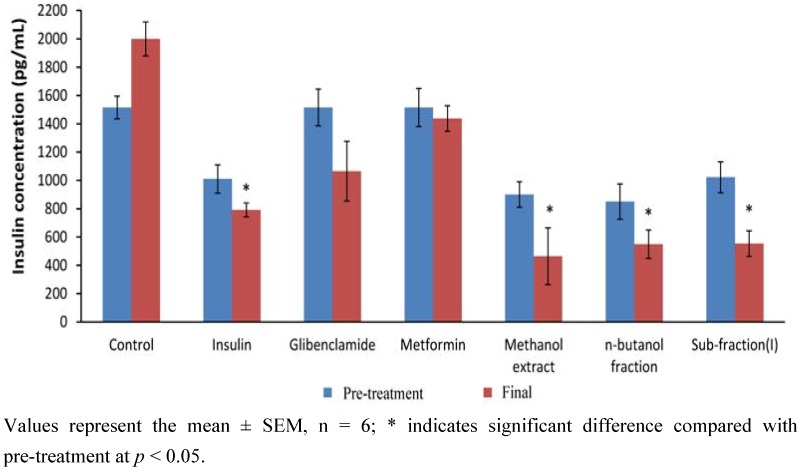
Plasma insulin concentrations of diabetic rats before and after treatment with methanol extract, *n*-butanol fraction and sub-fraction I and reference drugs (metformin, glibenclamide and insulin) for 12 days.

Effect of 12-day repeated administration of the most active extract (methanol), fraction (*n*-butanol) and sub-fraction (I) as well as positive controls (metformin, glibenclamide and insulin) on blood glucose and plasma insulin of diabetic rats is shown in Figure 5 and Figure 6, respectively. The results showed that the active extract, fraction and sub-fraction significantly decreased blood glucose after 12 days treatment compared both to diabetic control and concentration at the beginning of treatment (*p* < 0.05). The extent was also found to be slightly lower by PM extract/fraction/sub-fraction than metformin and glibenclamide. Measured plasma insulin concentration was significantly lowered in the methanol extract, *n*-butanol fraction and sub-fraction I treated groups compared to their respective concentrations at onset of experiment and diabetic control (*p* < 0.05). However plasma insulin concentration in the metformin and glibenclamide groups was only slightly reduced.

#### 2.1.6. Effect of Active Extract, Fraction and Sub-Fraction on *in Vitro* Intestinal Glucose Absorption

**Figure 7:**
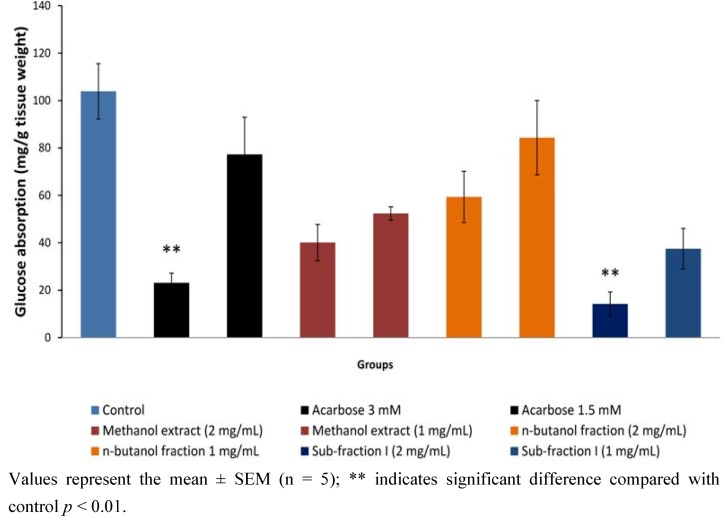
Effect of methanol extract, *n*-butanol fraction and sub-fraction I */* acarbose on *in vitro* intestinal glucose absorption by everted sac technique.

The effect of active *P. macrocarpa* extract and fracti on*/*acarboseon *in vitro* intestinal glucose absorption is shown in Figure 7. *P. macrocarpa* leaf extract and fractions showed a dose dependent inhibition effect on glucose absorption. At the two test concentrations (1 and 2 mg/mL), methanol extract and *n*-butanol fraction exerted 49.55% and 61.38%; and 18.78% and 42.84% inhibition on intestinal glucose transport relative to the normal control, respectively. These effects were however not statistically significant. The highest inhibition effect was shown by the *n*-butanol sub-fraction I (63.88 and 86.30% at 1 and 2 mg/mL, respectively, *p <* 0.05), which was correspondingly higher than the effect of acarbose, a standard α-glucosidase inhibitor (25.64% and 77.74%, *p <* 0.05). 

#### 2.1.7. Phytochemical Screening of Most Active Extract, Fraction and Sub-Fraction

**Figure 8:**
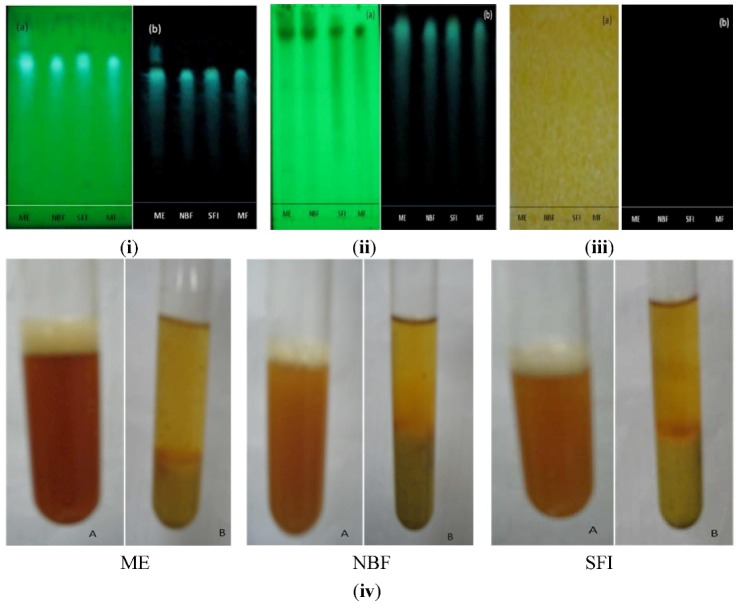
Qualitative phytochemical evaluation of the methanol extract (ME), *n*-butanol fraction (NBF) and sub-fraction I (SFI) of *P. macrocarpa* fruit pericarp, indicating the presence/absence of (**i**) flavonoids, (**ii**) terpenoids, and (**iii**) alkaloids on TLC chromatogram after spray with NP/PEG and detected under (a) visible or (b) UV light; and (**iv**) tannins determined by precipitation.

Phytochemical screening of the methanol extract, *n*-butanol fraction and sub-fraction I of *P. macrocarpa* fruit using thin layer chromatography (TLC) and/or test tube procedures revealed the presence of flavonoids, tannins and terpenoids, but absence of alkaloids (Figure 8). 

#### 2.1.8. Main Active Constituent in *P. macrocarpa* Fruit Pericarp

**Figure 9:**
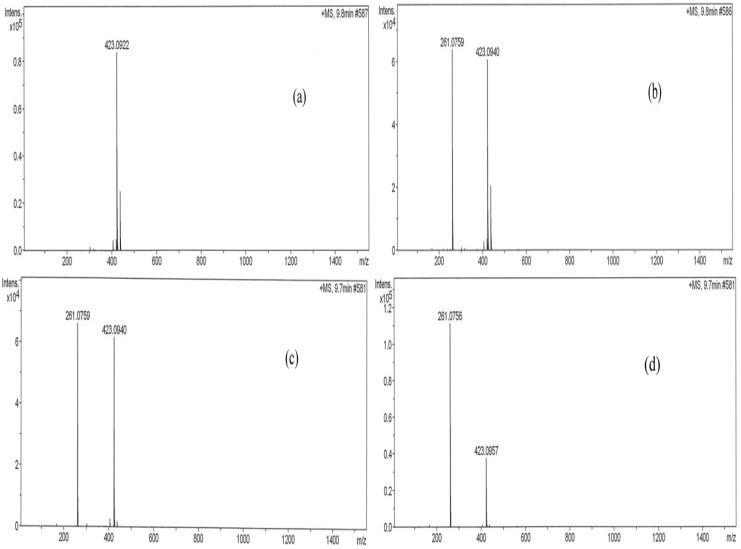
LC chromatogram of (**a**) standard mangiferin, (**b**) methanol extract, (**c**) *n*-butanol fraction and (**d**) sub-fraction I of *P. macrocarpa*. The analysis was performed on a HyStar LC model using an Acclaim Polar Advantage II 3 μm C18 column (2.1 × 150 mm). The mobile phase used consisted of (A) water-formic acid (99.9:0.1 v/v) and (B) CH_3_CN. The injection volume of each sample (1 mg/mL) was 20 μL. The flow rate of the mobile phase was set at 0.2 mL/min and peaks monitored at 340 nm.

Figure 9 shows LC-MS profiles of methanol extract, *n*-butanol fraction and sub-fraction I of PM fruits pericarp compared with a standard, mangiferin. The analyses which were performed at identical conditions of wavelength (340 nm) and retention time (30 min) identified mangiferin in the samples analysed, but in varying proportions. Calculation based on simple linear regression curve revealed that methanol extract, *n*-butanol fraction and sub-fraction I samples of *P. macrocarpa* pericarp contain 9.52%, 33.30% and 22.50% of mangiferin, respectively.

### 2.2. Discussion

The present study was in furtherance of the systematized ethno-medical approach to prospecting for antidiabetic agent(s) from the fruit pericarp of PM. Having previously demonstrated the methanol extract as the most active, it was further fractionated in the present investigation yielding four fractions —chloroform (5.45%), ethyl acetate (7.36%), *n*-butanol (33.36%) and aqueous (27%). These fractions were therefore tested for their effects on glucose tolerance in non-diabetic rats, and the result indicated that the *n*-butanol fraction was the most effective in responding to abrupt hyperglycemia. This *n*-butanol fraction significantly suppressed the peak blood glucose (PBG) just 15 min after intraperitoneal loading, similar to the effect of metformin. Hassan *et al.* [10] had earlier reported a similar observation for metformin and *Gynura procumbens,* an antidiabetic medicinal plant, where PBG was suppressed only 15 min after glucose load. PM may thus share at least in part the properties of *G. procumbens* as an antidiabetic plant. Plants or agents with this property of prompt response to glucose challenge possesses the needed potential for effective management of post-prandial hyperglycemia (PPH), a major concern in treatment of type 2 diabetes [11]. The potent anti-hyperglycemic action of the *n*-butanol fraction was further proven in a 12-day daily administration of the fractions in diabetic rats, by 66.67% reduction in glucose levels similar to metformin (51.11%), glibenclamide (66.67%) and insulin (71.43%), but no significant effect were shown by chloroform, ethyl acetate and water extracts. This result agrees in part with the earlier report of Triastuti *et al.* [4]. In this study, the methanol, ethyl acetate, *n*-butanol and water extracts of PM all lowered blood glucose after 14 days treatment in alloxan-induced diabetic animals. The difference is obviously traceable to the extraction procedure. Their procedure was not sequential, leaving a high probability of finding reasonable amount of the active components in all solvents used. The present investigation on the other hand, employed a sequential solvent extraction procedure (activity-guided), in which case the active glucose lowering components must have been extracted sequentially to concentrate in the *n*-butanol leaving less significaant amount for any observable antihyperglycemic effect in the other solvents. 

The *n*-butanol fraction was futher fractionated in dry-colum flash chromatography to obtain two sub-fractions, namely sub-fraction I (40%) and sub-fraction II (16%). Results of the effect of these sub-fractions on glucose tolerance in normal and single dose test on 7 h blood glucose in diabetic rats revealed SFI as containing the antidiabetic compounds in PM. The anti-hyperglycemic activity from the foregoing appeared correlated to the extract/fractions with the highest percentage yield in each case: methanol extract (18.55%), *n*-butanol fraction (33.36%) and SFI (40.00%). We therefore compared the effect of the identical dosage of these fractions on blood glucose and plasma insulin with positive controls, a typical biguanide (metformin), a sulfonylurea (glibenclimade) and insulin to verify the occurence of active components and to elucidate the possible the antidiabetic mechanism of PM. It was indicated from the result that, whereas all three fractions significantly reduced blood glucose after 12 days treatment, the extent of decrease did not correlate with the yield in the methanol extract, *n*-butanol fraction and sub-fraction I. However, dose-response studies (250 mg/kg, 500 mg/kg and 1,000 mg/kg) conducted separately with the individual fractions showed dependence on dosage for anti-hyperglycemic action (data not shown). Several active components including kaempferol, myricetin, naringin, and rutin have been isolated from fruit pericarp of *P. macrocarpa* [12]. These compounds may interact synergistically to exert the antidiabetic effect.

Plasma insulin concentration was significantly reduced by the methanol extract, *n*-butanol fraction and SFI, but not metformin and glibenclamide at end of the 12 day treatment. This observation is partly in accordance with the report of Luo *et al.* [13] on two novel antidiabetic compounds isolated from *Pycnanthus angolensis* namely SP-18904 and SP-18905. Similar to the effect of PM, these researchers observed a decline in plasma glucose concentration associated with lower plasma insulin concentrations in diabetic animals treated with the novel plant compounds. The simultaneous lowering in plasma glucose and insulin may indicate that the active compounds compounds of *P. macrocarpa* are not insulin secretagogues, but rather seem to be enhancing the ability of insulin to stimulate glucose disposal. Glibenclamide a known sulfonylurea, which acts by sensitizing insulin production and may have elicited insulin secretion from the residual or regenerated β-cells in diabetic rats, rather than causing a decrease. Coskun *et al.* [14] have earlier indicated weak insulin-immunoreactivity in a few β-cells in the islet of Langerhans four weeks after STZ treatment, implying possibility of finding residual β-cells long after STZ action. A novel mechanism of metformin action in glucose lowering in STZ diabetic rats involves an increase of β-endorphin secretion from adrenal glands to stimulate opioid β-receptor linkage, leading to an increase of GLUT-4 gene expression and an attenuation of hepatic PEPCK gene expression [15]. Exogenous β-endorphin are known to induce an increase of circulating insulin in humans with or without diabetes [16]. If this is true, then there is a high probability that the increased β-endorphins may have influenced the plasma insulin level in the present STZ diabetic model. PM may therefore constitute a plant for sourcing natural products or for standardization into dosage forms for management of type 2 diabetes, which accounts for about 90–95% of diabetic patients [17].

A phytochemical analysis was carried out to determine the nature of the active components present in the active fractions. This indicated tannins, terpenoids and flavonoids as the major groups of compounds. Handra *et al.* [12] have isolated four different types of flavonoids from PM with strong antioxidant and antimicrobial activities and Triastuti and Choi [18] have inplicated PM in modulation of oxidative stress in diabetes. Further, a current review of plant phytochemicals by Kumar *et al.* [19] has listed several examples of antidiabetic compounds belonging to tannins, flavonoids and terpenoids with extrapancreatic mechanisms. Moreover, in the present study, SFI was shown to exhibit a stronger inhibition than acarbose, a standard α-glucosidase inhibitor, against glucose transport/ absorption in an isolated rat intestine, a desired property for management of type 2 diabetes mellitus.

LC-MS analyses carried out in this study identified mangiferin as a major compound found in the active extract, fraction and sub-fraction. This identified bio-constituent may therefore be responsible for the antidiabetic activity of PM. Earlier studies had demonstrated that mangiferin possesses significant antidiabetic, antihyperlipidemic and antiatherogenic properties thus suggesting its beneficial effect in the treatment of diabetes mellitus associated with hyperlipidemia and related cardiovascular complications [22,23]

## 3. Experimental

### 3.1. Plant Material Collection and Preparation of Extracts

The dried fruit of PM were collected from Kepala Batas, Seberang Perai, Pulan Pinang, Malaysia. The pericarps of the fruits were sliced, dried, and ground into powder using a milling machine, and thereafter weighed and stored in air tight containers until use. About 2,400 g of the powder was sequentially extracted with petroleum ether (32 L), then methanol (32 L) using a Soxhlet apparatus (40 °C) for 48 h each. The residue from the methanol extraction after complete drying was re-extracted with water by maceration at 60 °C for 24 h. The extraction procedure with each solvent was repeated three times and the different extracts obtained were filtered with Whatman No. 1 filter paper and concentrated *in vacuo* by rotary evaporation (Buchi Laboratorium-Technik AG, Flawil, Switzerland) at reduced pressure. The concentrated extracts were frozen at −70 °C for 48 h then freeze dried under vacuum at −40 °C for 24 h yielding 73.6 g (3.06%), 445.36 g (18.55%) and 146 g (6.08%) of dried petroleum ether, methanol and aqueous extracts respectively. These were kept in the freezer from where aliquots were withdrawn for further testing.

#### 3.1.1. Fractionation of Methanol Extract

The methanol extract of PM was further fractionated by initially dissolving 110 g of the extract in 500 mL of water and mixed in a beaker. The suspension obtained was transferred into a 1 L separating funnel and extracted with 3 × 250 mL chloroform. The combined chloroform fraction was dried by using anhydrous sodium sulphate, followed by further concentration in a rotary evaporator. The aqueous layer was extracted with 3 × 250 mL ethyl acetate. The combined ethyl acetate fraction was washed with water, dried over anhydrous sodium sulphate and concentrated further with rotary evaporator. Finally, the aqueous layer was extracted with *n*-butanol 5 × 250 mL and the combined *n*-butanol fractions were concentrated in a rotary evaporator. The remainder aqueous fraction was also concentrated on a rotary evaporator. Concentrated fractions were kept in freezer at −70 °C for 24 h and thereafter freeze-dried at −40 °C for 24 h with the yield of 18 g (5.45%), 24.3 g (7.36%), 110.1 g (33.3%) and 89.1 g (27%) of chloroform, ethyl acetate, *n*-butanol and aqueous fractions, respectively.

#### 3.1.2. Further Fractionation of Active *n*-Butanol Fraction Using Dry-Column Flash Chromatography

A glass column chromatography (27 × 5 cm) fitted with a stopcock was used in the separation, and loaded with 100 g of silica gel (Merck, 7730) slurried in 300 mL petroleum ether. Vacuum suction was applied using a vacuum pump and the silica was carefully packed onto the flat bottom of the flask, initially moved around the circumference and progressed gradually towards the center. This is to ensure a totally leveled and well-compacted bed yield. Twelve (12 g) grams of the *n*-butanol fraction was pre-adsorbed onto silica gel adsorbent (200–400 mesh) by firstly solubilizing it in 100 mL of methanol, followed by addition of the silica gel (24 g) then mixing. Thereafter this mixture was subjected to rotary evaporation for drying until the mixture was completely dried. The dried extract-adsorbent mixture was then evenly loaded onto the top of the already packed column by applying suction. The column was first eluted with 2 × 300 mL 100% chloroform followed successively by 2 × 300 mL chloroform- methanol in graded ratios (9:1), (8:2), (7:3), (6:4), (5:5), (4:6), (3:7), (2:8), (1:9), (0:10) and finally with chloroform-methanol-water (7:13:2). Fractions (fixed volume) were collected into pre-labeled tubes and examined with thin layer chromatography using *n*-butanol/acetic acid/water (4:1:5) as the mobile phase. Fractions with similar profiles were pooled together to obtain two sub-fractions namely BFI and BFII. These were dried under vacuum and kept in the freezer −70 °C for 24 h then freeze dried to obtain 20 g (40%) and 8 g (16%) of sub-fractions I and II, respectively.

### 3.2. Animals

Healthy male Sprague Dawley (SD) rats weighing between 200–250 g and obtained from the Animal Research and Service Centre, Universiti Sains Malaysia (USM) were used in the study. The animals were housed and kept at 25–30 °C in the Animal Transit Room, School of Pharmaceutical Sciences, USM. They were allowed free access to food (standard laboratory chow, Gold Coin Sdn. Bhd., Malaysia) and tap water *ad libitum*. The experimental procedure was approved by the Animal Ethics Committee of Universiti Sains Malaysia (USM) Penang, Malaysia.

### 3.3. Induction of Diabetes

Diabetes was induced in rats by intraperitoneal injection of 65 mg/kg b.w. of streptozotocin (STZ, Sigma, Chemicals Co, St. Louis, MO, USA) reconstituted in normal saline, after an overnight fast [20]. Seventy-two (72) hours after streptozotocin administration, blood glucose level was measured with blood collected from tail vein puncture using an Accu-check Advantage II Clinical Glucose meter (Roche Diagnostics Co., Corporation 9115 Hague Read Indianapolis, IN, USA). Rats with fasting blood glucose ≥15 mmol/L were considered diabetic and included in the study.

### 3.4. Anti-Hyperglycemic Test with Fractions from Methanol Extract of *Phaleria macrocarpa*

#### 3.4.1. Intraperitoneal Glucose Tolerance Test (IPGTT) in Normal Rats

Thirty-six rats were divided into six groups consisting of six rats (n = 6) per group. After an overnight fast (but with free access to water), groups 1–4 were respectively treated with 1 g/kg body weight of chloroform, ethyl acetate, *n*-butanol and aqueous fractions of the methanol extract of PM. Groups 5 and 6 which served as the positive and normal controls respectively received metformin (250 mg/kg body weight) and normal saline. Sixty (60) min after oral treatment, 1 g/kg glucose was administered intraperitoneally to all the rats, and glucose was measured in blood samples obtained via tail vein puncture at −60 min (just before the extract was administered), 0 min (prior to the glucose load) and at 15, 30, 45, 60, 90 and 120 min post glucose load [10]. 

#### 3.4.2. Short-Term (12 days) Anti-Hyperglycemic Test in STZ-Induced Diabetic Rats

Forty eight (48) diabetic rats were assigned equally into eight groups of six rats. Group 1, the control group, received normal saline, groups 2–4 constituting the positive controls were respectively treated with 5 units/kg insulin (s.c.), 10 mg/kg glibenclamide (p.o.) and 250 mg/kg metformin (p.o.), whereas groups 5–8 received p.o. 1 g/kg body weight of chloroform, ethyl acetate, *n*-butanol and aqueous fractions of methanol extract of PM respectively. Treatment was once a day, and lasted for 12 days. Blood glucose was measured in blood collected via tell vain on days 3, 6, 9 and at the end of study (12th day) using the Accu-check Advantage II. 

### 3.5. Anti-Hyperglycemic Test with n-Butanol Sub-Fraction of *Phaleria macrocarpa*

#### 3.5.1. Glucose Tolerance Test (IPGTT) in Non Diabetic Rats

The procedure was as in section 2.5i above, but in this test 1 g/kg body weights each of the *n*-butanol sub-fractions I and II was administered instead of the methanol fractions.

#### 3.5.2. Acute/Single Dose Glucose Response Test in Streptozotocin Diabetic Rats

In this test, thirty diabetic rats were randomly categorized into five groups (n = 6). After an overnight fast, Group 1, the control, was treated with normal saline, groups 2 and 3, were given 1 g/kg body weights of sub-fractions I and II respectively, and groups 4 and 5, which served as the positive controls, received 10 mg/kg glibenclimide p.o. and 5 unit/kg insulin s.c. respectively. Blood was collected from tail vein before (0 min) and at 1, 2, 3, 5 and 7 h post treatment for glucose measurement using Accu-check Advantage II clinical glucose meter.

### 3.6. Anti-Hyperglycemic Test of the Most Active Extract/Fraction/Sub-Fraction

To verify the effect of active components, 42 diabetic and six normal rats were assigned into eight groups (n = 6) and separately treated with the most active fractions including methanol extract, *n*-butanol fraction and sub-fraction 1 concurrently with positive controls for 12 days. Group 1, the diabetic control, received saline treatment; groups 2–4, the positive controls were treated respectively with 250 mg/kg metformin (p.o.), 10 mg/kg glibenclimide (p.o.) and 5 units/kg insulin (s.c.), and groups 5–7 received 1 g/kg (p.o.) each of methanol extract, *n*-butanol fraction and sub-fraction I of PM, respectively. The choice dose of 1 g/kg was determined from a preliminary dose-response study carried out in our laboratory (results not shown). Treatment was once per day and blood samples collected at the onset and the end of study were used for glucose and plasma insulin measurements. 

### 3.7. Plasma Insulin Determination

The blood samples collected into hematocrit-capillary tubes (Hirschmann Laborgerate GmbH & Co. KG, Eberstadt, Germany) were centrifuged at 12,000 rpm for 3 min, after 60 min. The plasma separated was stored at −20 °C for the measurement of insulin. The insulin was assayed by enzyme-linked immunosorbent assay (ELISA) using the rat anti-nsulin ELISA Kit (Crystal Chem, Corporate Headquarters 1536 Brook Drive, Suite A Downers, IL, USA). Briefly, microwells coated with a mixture of highly purified preparations of bovine, porcine and recombinant human insulin followed by blocking the unreacted sites to reduce non-specific binding, react with antibodies specific to insulin present in controls, calibrators and plasma samples, by binding to the coated antigen. The Antigen-Antibody complex is reacted with enzyme (horseradish peroxidase, HRP) labeled anti rat IgG conjugate resulting in the anti-insulin antibodies being sandwiched between the solid phase antibody and the enzyme conjugate. The enzyme then converts an added substrate (3,3,5,5-tetramethylbenzidine, TMB) to form a coloured substance, whose colour intensity is proportional to the concentration of antibodies present in the samples at 450 nM.

### 3.8. Measurement of Glucose Absorption in Isolated Rat Intestine

Sprague Dawley rats (150–200 g) were sacrificed after an overnight fast and their abdominal walls dissected. The jejunum (21 cm from the pylorus) was isolated and cut into segments of 5 cm long. These segments were everted and suspended in oxygenated tyrode solution (342 mM NaCl, 6.7 mM KCl, 5.9 mM CaCl_2_·2H_2_O, 5.3 mM MgCl_2_, 59.5 mM NaHCO_3_, 2.08 mM NaH_2_PO_2_ and 5.5 mM glucose). The everted segments were then tied securely with cotton thread at both ends with the inside filled with 0.5 mL of tyrode solution forming sacs. The sacs were then incubated in the presence of the test substances at 37 °C for 60 minutes in 15 mL test tube baths gassed with 95% O_2 _and 5% CO_2_. The test substances were methanol extract (1 and 2 mg/mL), *n*-butanol fraction (1 and 2 mg/mL) and sub-fraction I (1 and 2 mg/mL) of *P. macrocarpa.* Acarbose, an α-glucosidase inhibitor (1.5 and 3 mM) and tyrope solution were used as positive and negative controls respectively. Glucose concentration in the incubation test tube bath before and after incubation was measured using a glucose analyzer (YSI model 23A Sidekick®, Yellow Springs, OH, USA). Amount of glucose absorbed or transported was calculated as the difference in glucose concentration before and after incubation thus: [(Gb – Ga) / Wi] where Wi = weight of intestinal segment (g); Gb = glucose concentration before incubation; Ga = glucose concentration after incubation.

### 3.9. Phytochemical Screening of the Active Extract, Fraction and Sub-Fraction

Tests for the presence of selected phyto-compounds in PM—Flavonoids, terpenoids, alkaloids and tannins were carried out for the most active extract (methanol), fraction (*n*-butanol) and sub-fraction (I) in comparison with the reference compound (mangiferin) using specific reagents (to develop the chromatograms) on thin layer chromatography [21]. 

### 3.10. LC-MS Analysis of the Methanol Extract, *n*-Butanol Fraction and Sub-Fraction I

The chemical composition of the methanol extract, *n*-butanol fraction and sub-fraction I analysed using a HyStar LC (Bruker Daltonik GmbH, Bremen, Germany), Acclaim Polar Advantage II C18 column (2.1 mm × 150 mm, 3 µm i.d.), and an auto-sampler at 35 °C assay temperature. The binary mobile phase used consisted of (A) water-formic acid (99.9:0.1 v/v) and (B) CH_3_CN. The gradient elution program was set to 10–90% B (0–5 min), 90–10% B (5–15 min), 90–10% A (15–25 min) and 10–90% A (25–30 min). The standard mangiferin and samples were prepared at a concentration of 1 mg/mL in the mobile phase. Before injection, the samples were filtered through a polytetraflouroethylene (PTFE) membrane. The injection volume of each sample (1 mg/mL) was 20 µL. The flow rate of the mobile phase was set at 0.2 mL/min and peaks monitored at 340 nm. 

### 3.11. Statistical Analysis

All data were expressed as the mean ± SEM. Statistical analysis of data was performed using one-way analysis of variance (ANOVA) followed by Dunnett test for post hoc analysis. *P* < 0.05 and *p <* 0.01 were considered as significant.

## 4. Conclusions

In conclusion, a flavonoid-rich sub-fraction obtained from fruit pericarp of PM by activity-guided fractionation, was found to demonstrate the most potent antidiabetic activity. This fraction was found via LC-MS analysis to contain 22.5% mangiferin, a typical flavonoid whose potent antidiabetic action had been reported by other researchers [22,23]. In an *in vitro* model, this fraction was found to exert a more potent effect than acarbose in inhibiting rat intestinal glucose transport/absorption. Hence, the antidiabetic action of PM may be exerted by extra-pancreatic mechanisms. Further study is however necessary to isolate and characterize the active compound(s) in SFI and elucidate in detail the antidiabetic mechanism(s).
